# Giant phonon anomalies in the proximate Kitaev quantum spin liquid α-RuCl_3_

**DOI:** 10.1038/s41467-021-23826-1

**Published:** 2021-06-10

**Authors:** Haoxiang Li, T. T. Zhang, A. Said, G. Fabbris, D. G. Mazzone, J. Q. Yan, D. Mandrus, Gábor B. Halász, S. Okamoto, S. Murakami, M. P. M. Dean, H. N. Lee, H. Miao

**Affiliations:** 1 Materials Science and Technology Division, Oak Ridge National Laboratory, Oak Ridge, TN USA; 2 Department of Physics, Tokyo Institute of Technology, Okayama, Meguro-ku, Tokyo Japan; 3 Tokodai Institute for Element Strategy, Tokyo Institute of Technology, Nagatsuta, Midori-ku, Yokohama, Kanagawa Japan; 4 Advanced Photon Source, Argonne National Laboratory, Argonne, IL USA; 5 Condensed Matter Physics and Materials Science Department, Brookhaven National Laboratory, Upton, NY USA; 6 Laboratory for Neutron Scattering and Imaging, Paul Scherrer Institut, Villigen, Switzerland; 7 Department of Materials Science and Engineering, the University of Tennessee at Knoxville, Knoxville, TN USA

**Keywords:** Quantum fluids and solids, Magnetic properties and materials

## Abstract

The Kitaev quantum spin liquid epitomizes an entangled topological state, for which two flavors of fractionalized low-energy excitations are predicted: the itinerant Majorana fermion and the Z_2_ gauge flux. It was proposed recently that fingerprints of fractional excitations are encoded in the phonon spectra of Kitaev quantum spin liquids through a novel fractional-excitation-phonon coupling. Here, we detect anomalous phonon effects in α-RuCl_3_ using inelastic X-ray scattering with meV resolution. At high temperature, we discover interlaced optical phonons intercepting a transverse acoustic phonon between 3 and 7 meV. Upon decreasing temperature, the optical phonons display a large intensity enhancement near the Kitaev energy, *J*_K_~8 meV, that coincides with a giant acoustic phonon softening near the Z_2_ gauge flux energy scale. These phonon anomalies signify the coupling of phonon and Kitaev magnetic excitations in α-RuCl_3_ and demonstrates a proof-of-principle method to detect anomalous excitations in topological quantum materials.

## Abstract (web summary)

It was recently proposed that the coupling between phonons and fractional excitations of a Kitaev quantum spin liquid can be detected in its phonon dynamics. Here, the authors report signatures of this coupling, manifested in low-energy phonon anomalies measured by inelastic X-ray scattering with meV resolution.

## Introduction

In correlated quantum materials, the nature of electronic interactions and their ground state topology is intimately linked to the geometry of the underlying lattice^1–6^. The low-energy excitations arising from pure electronic degrees of freedom inevitably interact with the crystal lattice, leaving behind their fingerprints in the phonon spectrum. Hitherto, the interactions of phonons with “conventional” quasiparticles of either Bose–Einstein or Fermi–Dirac statistics, such as magnons in magnets^7^, phasons and amplitudons in density waves^8–10^ and Bogoliubons in superconductors^11^, have been explored extensively. In contrast, the coupling between phonons and fractional excitations, including spinons in one-dimensional magnets^1–3,12–15^, and Majorana fermions (MFs) and Z_2_ gauge fluxes that are thought to exist in the Kitaev quantum spin liquids (QSL)^16–25^, have remained elusive. The discovery of such fractional-excitation-phonon coupling (FPC) is of fundamental importance, as they carry key information of the intertwined quantum state^12–15^. In particular, the coupling of phonons to the itinerant MFs has been predicted to play a pivotal role in the realization of the field-induced quantum thermal Hall effect in α-RuCl_3_^26–29^, which is a signature of quantum entanglement in Kitaev-QSLs^30–32^.

**Fig. 1:**
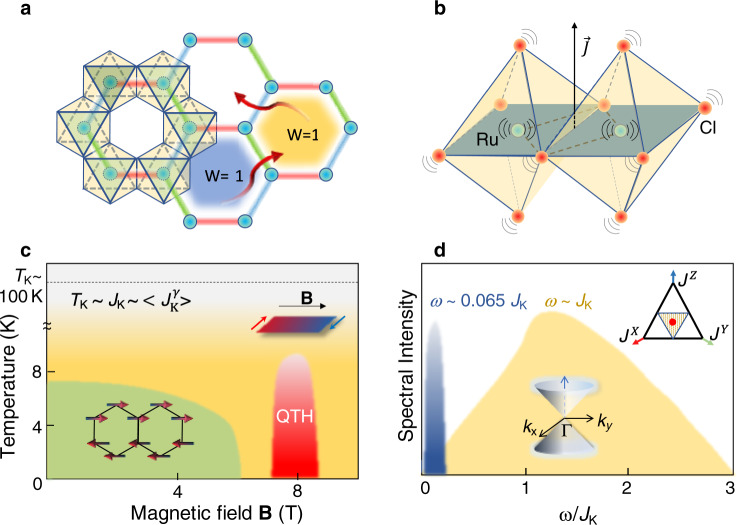
Schematics of a Kitaev-QSL and the phase diagram of α-RuCl_3_. **a** Structure motif of α-RuCl_3_ based on a honeycomb lattice of edge-sharing Ru–Cl octahedra. The red, green, and blue bonds represent three orthogonal Kitaev interactions ${J}_{K}^{\,\gamma }$. In the pure Kitaev model [Eq. (1)], the low-energy excitations fractionalize into itinerant MFs (red arrows) and anyonic Z_2_ flux (blue and yellow hexagons) W = ±1 represents the Z_2_ index. **b** The nearly 90° Ru–Cl–Ru bonds and the moderate spin–orbit-coupling favor an Ising-type magnetic interaction that is perpendicular to the Ru–Cl–Ru plane highlighted in a blue–green color. Lattice vibrations perturbatively modify the magnetic interactions, which induce a coupling between phonons and fractional excitations. **c** illustrates the phase diagram of α-RuCl_3_ on a logarithmic-scale: below *T*_K_ ~ *J*_K_ ~8 meV (the yellow area) the thermal Hall conductivity, *κ*_xy_, becomes finite, indicating a proximate Kitaev-QSL with MF and Z_2_ gauge flux. In the green area (*T* < *T*_N_ =7 K), non-Kitaev terms drive the system into zigzag antiferromagnetic order. Under an external magnetic field (**B** > 7 T) that completely suppresses the magnetic order, the system is driven into a quantum thermal Hall state at finite temperature (the red area). **d** schematically shows two characteristic Kitaev energy scales in the isotropic limit: the itinerant MF excitation^18^ (yellow area) that is broadly peaked around *J*_K_ and the Z_2_ gauge flux excitations (blue area) near 0.065 *J*_K_.

Numerous studies have shown that the low-temperature phase of α-RuCl_3_ is a promising Kitaev-QSL candidate^17–24,30–32^. As displayed in Fig. 1a, the edge-sharing Ru–Cl octahedra form an effective spin-1/2 honeycomb network. The destructive quantum-interference through the close-to-90° Ru–Cl–Ru bonds significantly suppresses the Heisenberg magnetic exchange interaction, yielding a dominant Ising-type interaction (**J**) perpendicular to the Ru–Cl–Ru plane^33^ (Fig. 1b). Figure 1c schematically depicts the phase diagram of α-RuCl_3_. At zero magnetic field, the low-energy excitations in the paramagnetic phase are primarily determined by the Kitaev term^3,4,17–24,30–32^

1 $$H=\mathop{\sum}\limits_{\gamma ,\, <\, i,j \,> \, }{J}_{K}^{\,\gamma }{S}_{i}^{\gamma }{S}_{j}^{\gamma }$$

Here $${J}_{K}^{\,\gamma }\,$$($$\gamma =X,Y,Z$$) is the bond-dependent coupling parameter, and $$< i,j > $$ stands for nearest-neighbor pairs of spins at one of the *X, Y*, or *Z* bonds. The two characteristic energy scales are shown in Fig. 1d for the isotropic limit ($${J}_{K}^{\,\gamma }={J}_{K}$$). Below the Kitaev temperature scale $${T}_{k} \sim {J}_{K}$$, the low-energy excitations of Eq. (1) start to fractionalize into itinerant MFs and fluctuating Z_2_ gauge fluxes^34^. The former features a continuum that peaks broadly near *J*_K_, while the latter is a local excitation with an energy around 0.065 *J*_K_^16,20,25,34^. Below *T*_N_ = 7 K, non-Kitaev interactions such as remnant Heisenberg magnetic exchange couplings, stabilize zigzag antiferromagnetic order that is suppressed under magnetic field^35–39^. Above **B** ~7 T, a quantized thermal Hall conductivity (red region in Fig.1c) is observed, indicating strongly an entangled topological phase^30–32^. However, unlike the quantum Hall effect of electrons, it has been theoretically predicted that the quantum thermal Hall effect can only be approximate and requires strong FPCs^26–29^. Here we report experimental signature of the FPC in α-RuCl_3_ by uncovering two-types of phonon anomalies at zero magnetic field: a 35% enhancement of the phonon spectral weight near the Kitaev energy *J*_K_, and a giant phonon softening of ~15% below 2 meV^26–29^.

## Results

**Fig. 2:**
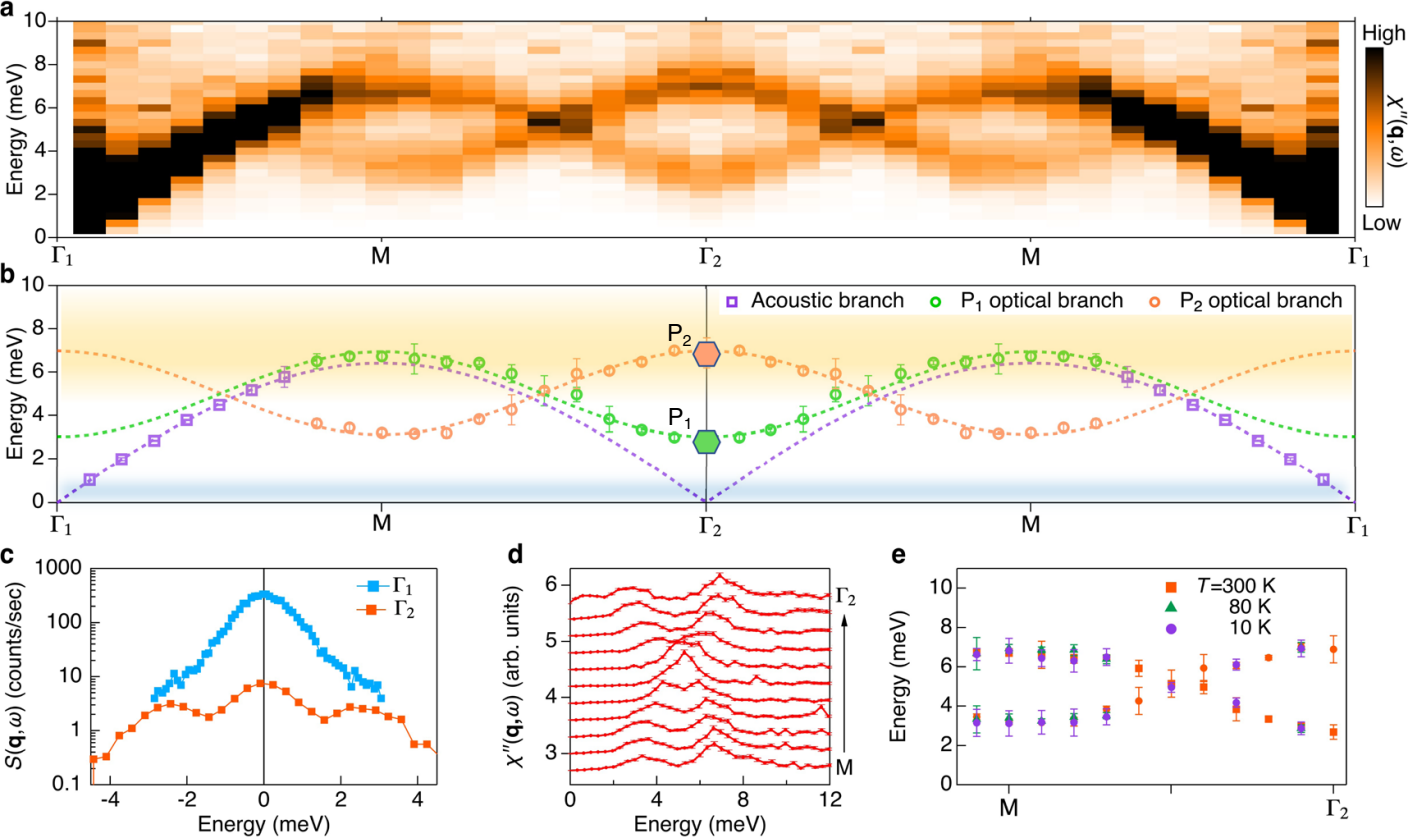
Room temperature phonon excitations in α-RuCl_3_. **a** Low-energy phonon excitations determined by IXS along the Γ_1_ (6, −3, 0)–M (6, −2.5, 0)– Γ_2_ (6, −2, 0) direction. The plot shows the Bose-factor corrected IXS intensity. The extracted peak positions are presented in (**b**) revealing interlaced optical phonons intercepting with the transverse acoustic phonon branch. The optical phonon energies at Γ_2_ are denoted by the green (P_1_) and orange (P_2_) hexagons, which are consistent with the phonon modes previously found by THz-spectroscopy^38^. The yellow and blue shaded areas correspond to the two characteristic Kitaev energy scales displayed in Fig. 1d. **c** IXS spectra at Γ_1_ and Γ_2_. The intensity is shown on a logarithmic scale. Note that due to the large intensity difference at Γ_1_ and Γ_2_, the acoustic phonon intensity is expected to be extremely weak near Γ_2_. **d** Constant momentum transfer cuts around the phonon-crossing. The two optical branches cross each other without imposing a hybridization gap. **e** The extracted phonon peak positions from M to Γ_2_ at different temperatures reveal a temperature-independent massless Dirac-cone. The error bars in **b**, **e** denote the 2*σ* returned from the fittings (see Supplementary Note 2). The error bars in **d** represent one standard deviation assuming Poisson counting statistics.

Figure 2a shows the imaginary part of the dynamical phonon susceptibility $\chi {\prime\prime} ({\bf{Q}},\omega )$ along Γ_1_(6, −3, 0)−M(6, −2.5, 0)−Γ_2_(6, −2, 0) in reciprocal lattice units (r.l.u.) at room temperature (see Supplementary Note 6 for first-principles calculations of phonon dispersion). The dynamical susceptibility is given by the fluctuation-dissipation theorem via $\chi {\prime\prime} ({\bf{Q}},\omega )=S({\bf{Q}},\omega )\times (1-{e}^{-\omega /{k}_{B}T})$, where $S({\bf{Q}},\omega )$ is the dynamical phonon structure factor that is directly measured by inelastic x-ray scattering (IXS). The total momentum transfer **Q** = **q** + **G***,* is composed of the reduced momentum transfer in the first Brillouin zone **q** and the reciprocal lattice vector **G**. The elastic contribution at *ω* = 0 was subtracted by fitting the IXS raw data in the entire energy window (see Supplementary Note 1 and Note 2). We selectively probe in-plane transverse phonon modes, whose dispersions (open circles and open squares) and sinusoidal fits (dashed curves) are shown in Fig. 2b. As shown in Fig. 2a, the intensity of the transverse acoustic phonon changes significantly from Γ_1_ to Γ_2_, reflecting their different Bragg peak intensities that are plotted in Fig. 2c. Two low-energy optical phonons, P_1_ and P_2_, are observed at the Brillouin zone center Γ_2_, corresponding to ${\omega }_{1}=2.7$ and ${\omega }_{2}=7$ meV, which are in good agreement with previous optical and neutron studies^19,40^. The two optical phonons carry opposite phonon velocities and form an interlaced structure that intercepts the acoustic phonon. An apparent phonon crossing occurs between Γ and M (Fig. 2d and e), suggesting possible Dirac-cone and topological phononic nodal-lines^41,42^.

**Fig. 3:**
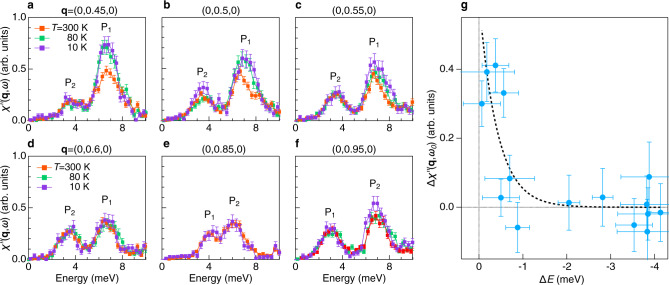
Itinerant MF-phonon coupling near *ω* ~ *J*_K_. **a**–**f** Spectra of the interlaced optical phonons at different reduced momentum transfer **q**. Here, we define **q** = (0, 0, 0) and (0, 1, 0) as Γ_1_ and Γ_2_, respectively, where the M point is at **q** = (0, 0.5, 0). The labels P_1_ and P_2_ denote the two optical phonon branches. Note the relative peak position of P_1_ and P_2_ switches at **q** = (0, 0.75, 0). The temperature dependent ${\chi }^{\prime\prime}({\bf{q}},\omega )$ shows a spectral weight enhancement at $\omega \sim {J}_{K}$ at low temperature. In **b** we notice a shoulder on P_2_ that may come from the acoustic mode. **g** The difference in the integrated phonon spectral weight, $\Delta \chi {\prime\prime} ({\bf{q}},{\omega }_{0})$, between 10 and 300 K as a function of$\,\Delta E={\omega }_{0}-{\omega }_{max}$. Here ${\omega }_{0}$ is the phonon peak position, ${\omega }_{max}$ = 7 meV is the band top of the interlaced optical phonons. The drastic increase of $\Delta \chi {\prime\prime} ({\bf{q}},{\omega }_{0})$ is fitted to an exponential function (dashed line). The vertical error bars in all panels represent one standard deviation based on Poisson counting statistics. The horizontal error bars in **g** denote the 2*σ* returned from the fitting algorithm that extract the spectral peak positions.

In α-RuCl_3_, *J*_K_ is estimated to be 5–9 meV in the low-temperature phase below 150 K^4,17–25,30^ (more discussions in Supplementary Note 5), which roughly corresponds to the top of the P_1_–P_2_ phonon band. Thus, if Majorana–phonon coupling is present, phonon anomalies are expected in the energy range shown in Fig. 2b. Moreover, a recent theoretical study of the pure Kitaev model predicts that the Majorana–phonon coupling is momentum dependent and peaks near the M and K point^28^. To uncover the energy and momentum-dependent coupling between the optical phonons and the suggested MFs, we compare the temperature-dependent $\chi {\prime\prime} ({\bf{q}},\omega )$ along the M–Γ_2_ path. A large spectral enhancement can be observed clearly in Fig. 3a–f. Near the M point, the peak intensity of P_1_ increases dramatically upon cooling from 300 K to 10 K. In contrast, the peak intensity of P_2_ is unchanged except the 10 K data at the M point. When approaching the Γ_2_ point (towards larger |**q**|), the intensity enhancement first decreases near the crossing-point (P_1_ and P_2_ crossed at *q* = 0.75), but then reappears at P_2_, which is higher in energy near the Γ_2_ point. Interestingly, we find that the spectral enhancement is different between the symmetry related points *q* = 0.45 and *q* = 0.55. As we show in Fig. 2a, the transverse acoustic phonon starts to merge with the optical phonon near the M point. Since the acoustic phonon intensity is stronger at *q* = 0.45, the asymmetric intensity enhancement suggests that the Majorana–phonon coupling is larger on the acoustic mode than the optical mode near $\omega \sim {J}_{K}$. To quantitatively show the spectral enhancement effect, we extract the temperature-induced difference in the integrated phonon intensity, $\Delta \chi {\prime\prime} ({\bf{q}},{\omega }_{0})={\int }_{{\omega }_{0}-\infty }^{{\omega }_{0}+\infty }[\chi {\prime\prime} ({\bf{q}},\omega ,10{\rm{K}})-\chi {\prime\prime} ({\bf{q}},\omega ,300{\rm{K}})]d\omega$, and plot $\Delta \chi {\prime\prime} ({\bf{q}},{\omega }_{0})$ as function of $\Delta E={\omega }_{0}-{\omega }_{{{\max }}}$ in Fig. 3g. Here ${\omega }_{0}$ denotes the phonon peak position and ${\omega }_{max}=7$ meV is the band-top energy of P_1_ and P_2_. Unlike the broad continuum observed in the spin correlation function^17–19,21–23^, $\Delta \chi ({\bf{q}},{\omega }_{0})$ decreases rapidly as ${\omega }_{0}$ moves away from *J*_K_ (Fig. 3). It also shows strong momentum dependence with the enhancement occuring around the high symmetry points M and Γ_2_ (see Supplementary Fig. 9). This observation is in qualitative agreement with theoretical calculation that shows energy and momentum dependent Majorana–phonon coupling^28^ (spectrum near the K point with spectral peak at higher energy is shown in Supplementary Fig. 6). The observed phonon enhancement is also consistent with a recent study of frustrated magnetic systems, which predicts large IXS cross-section for magnetic excitations^7^. We note, however, a quantitative understanding of the energy and momentum-dependent optical phonon enhancement may require theoretical calculations beyond the pure Kitaev model.

**Fig. 4:**
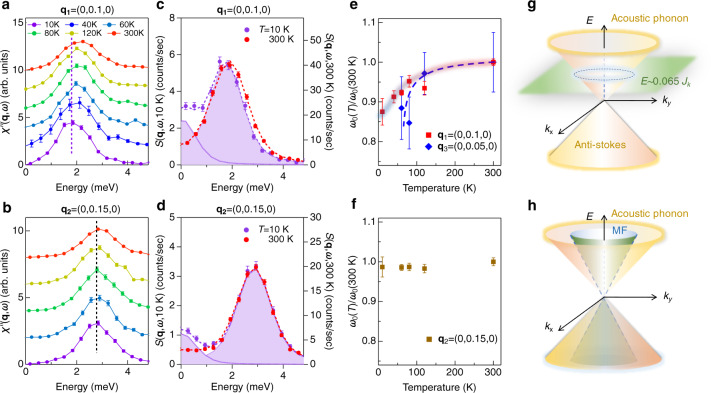
Giant acoustic phonon softening. **a**, **b** Show the temperature-dependent $\chi {\prime\prime} ({\bf{q}},\omega )$ at **q**_**1**_ = (0, 0.1, 0) in reciprocal lattice units (r.l.u.), *ω* ~2 meV and **q**_**2**_ = (0, 0.15, 0) r.l.u., *ω* *~*3 meV, respectively. **c** direct comparison of the IXS raw data, $S({{\bf{q}}}_{{\bf{1}}},\omega ),$ at *T* = 10 and 300 K. **d** shows the same plot as (**c**) but at **q**_**2**_. There is an apparent phonon softening at **q**_**1**_, while at **q**_**2**_, the effect is negligible. **e**, **f** The relative peak shift at **q**_**1**_, **q**_**2**_ and **q**_**3**._ The ~13% phonon softening at **q**_**1**_ (red squares in **e**) corresponds to a ~0.3 meV phonon peak shift. This value is as large as some well-known electron–phonon coupled systems^10^. The blue diamonds in **e** represent the relative peak shifts at **q**_**3**_ = (0,0.05,0) that show even larger softening-effect (~15% at 60 K), whereas **q**_**2**_ displays negligible change as shown in (**f**). This acoustic phonon anomaly, together with the spectral enhancement discussed in Fig. 3, present a full picture of the FPC in α-RuCl_3_. **g**, **h** Schematically show two phonon coupling mechanisms. **g** The flatband of the Z_2_ flux mode intercepts the acoustic phonon near *ω* ~0.065*J*_K_. **h** The nearly identical linear dispersion of the itinerant MF and the acoustic phonon at ${\bf{q}}\to 0$ causes a phonon renormalization at low temperature. The error bars in **a**–**d** represent one standard deviation assuming Poisson counting statistics. The error bars in **e**, **f** denote the 2*σ* returned from the fitting.

We then turn to the transverse acoustic phonon near Γ_1_. Figure 4a and b show the temperature-dependence of $\chi {\prime\prime} ({\bf{q}},\omega )$ at **q**_**1**_ = (0, 0.1, 0) (or **Q**_**1**_ = (6, −2.9, 0)) and **q**_**2**_ = (0, 0.15, 0) (or **Q**_**2**_ = (6, −2.85, 0)), respectively. At **q**_**1**_, the phonon peak position gradually shifts to lower energies. In contrast, it remains nearly unchanged at **q**_**2**_. The softening-effect is confirmed by directly comparing the raw data, $S({\bf{q}},\omega )$, at 10 and 300 K (Fig. 4c and d). The peak position is softened by about 13% at **q**_**1**_, which corresponds to ~0.3 meV shift in energy. Figures 4e and f show the relative peak shift ${\omega }_{0}(T)/{\omega }_{0}(300\,{\rm{K}})$ at **q**_**1**_ and **q**_**2**_ as function of temperature. We find that the acoustic phonon softening at **q**_**1**_ becomes progressively stronger below 80 K, consistent with the thermal Hall effect in α-RuCl_3_ where the thermal Hall conductivity, *κ*_xy_, starts to increase. In Fig. 4e, we further show the phonon softening at **q**_**3**_ = (0, 0.05, 0). The error-bars returned from fittings are larger at **q**_**3**_ as the elastic intensity becomes stronger when approaching the Bragg peak. Interestingly, the relative phonon softening at **q**_**3**_ (~15%) is even larger when compared to **q**_**1**_. This suggests an enhanced renormalization for long wavelength acoustic phonons.

## Discusson

The discovery of temperature and energy dependent phonon softening provides important information on the FPC in α-RuCl_3_. In the pure Kitaev model [Eq. (1)], quantum fractionalization occurs at *T*_K_ ~ *J*_K_ ~100 K^43^, in agreement with our observations. Below *T*_K_, the dispersionless gauge flux excitation crosses the linear dispersing acoustic phonon near $\omega =0.065{J}_{K} \sim 0.5$ meV^23^ and induces a phonon anomaly near this energy scale (Fig. 4g). The observation of enhanced phonon softening [$\omega ({{\bf{q}}}_{1})=2$ meV and $\omega ({{\bf{q}}}_{3})=$ 1 meV as $\omega \to 0.065{J}_{K}$ is consistent with this picture, where the softening effect is expected to be significantly suppressed for $\omega ({\bf{q}})\gg 0.065{J}_{K}$. Figure 4h depicts another scenario that attempts to explain the phonon-softening. Here, the acoustic phonon and the itinerant MFs possess nearly identical linear dispersions at ${\bf{q}}\to 0$^29^. This enhances Majorana–phonon coupling that yields a renormalization of the phonon dispersion below *T*_K_^28,29^. To justify this conjecture, we extract the acoustic phonon velocity ${v}_{{\rm{ph}}} \sim$16 meV Å ($\hslash =1$), which is based on the room-temperature phonon dispersion shown in Fig. 2. In the isotropic limit^16^, the velocity of the itinerant MF is ${v}_{{\rm{MF}}}=\frac{\sqrt{3}}{4}{J}_{K}a$, where the in-plane lattice constant $a=5.9639$ Å. Comparing ${v}_{{\rm{ph}}}$ and ${v}_{{\rm{MF}}}$ gives ${J}_{K}$ ~ 6.2 meV, comparable to the experimental value. Besides the Z_2_ gauge flux and MFs, in more realistic models with non-Kitaev interactions^44,45^, other fractional excitations may also be consistent with the observed phonon anomalies. It is important to note that the charge and magnetic excitations below 2 meV still remain unresolved in α-RuCl_3_. In particular, direct experimental evidence of Z_2_ gauge flux is not well established yet. The observed acoustic phonon softening below 2 meV demonstrate a small energy scale in α-RuCl_3_ that strongly renormalizes the acoustic phonon spectrum and hence may be responsible for the quantized thermal Hall effect.

Finally, we discuss the possibility of magnon–phonon coupling. Below *T*_N_, a gapped magnon excitation between 2–7 meV was observed in α-RuCl_3_ by previous neutron studies^19,21,22,37^. However, as we show in Figs. 3 and 4, the phonon anomalies onset at *T*_K_, which is well above *T*_N_. More importantly, evidence of an enhanced phonon softening is observed at *ω* =1 meV (see **q**_**3**_ in Fig. 4e and Supplementary Fig. 5), which is well below the magnon gap. Therefore, a magnon–phonon coupling is unlikely giving rise to the observed acoustic phonon softening. However, the magnon–phonon coupling may indeed be present in α-RuCl_3_. As we show in Fig. 2, the P_2_ phonon energy is the same as the magnon energy near the M point^19,21,38^. Interestingly, the P_2_ phonon intensity at the M point is enhanced at 10 K~*T*_N_, supporting magnon–phonon coupling^46^. In addition, strong anharmonicity is proposed in the magnon excitation of this material^47^, which represents the break-down of the spin quasiparticles. Such excitations contain extremely broad features^47^ that are contradictory to the well-defined energy scale of the phonon anomalies observed here.

Our discovery of two-types of phonon anomalies, i.e., the spectral enhancement in the optical phonon and the acoustic mode softening, provides experimental signature of FPC in the proximity of Kitaev-QSL^26,27^. Beyond the aforementioned implications, our observation has an even deeper impact on correlated topological quantum states. First of all, our approach can be immediately applied to other Kitaev-QSL candidates^1,3,4^, such as iridates^4,48^, where the inelastic neutron scattering experiments are difficult to perform due to strong neutron absorption of Ir. Moreover, it has been predicted that in *U*(1) spin liquids the spinon Fermi surface features a large singularity at 2**k**_F_, which induces phonon anomalies at **q** = 2**k**_F_^12^. Both kagome and triangular lattices have been speculated to host such charge neutral Fermi surfaces^49,50^. More recently, a giant thermal Hall effect has been observed in the cuprate high-*T*_c_ superconductors^13^ with large phonon contributions^51^. While mechanisms based on chiral spin liquid or topological spinons^14,15^ have also been proposed, the theoretically predicted *κ*_xy_ is 50% smaller than the experimental value^14^, suggesting large phonon effect. Our observation of FPC in α-RuCl_3_ validates phonons as a sensitive probe to uncover hidden fractional and non-local excitations, and hence can help resolving key puzzles in correlated and entangled quantum states.

## Methods

### Sample preparation and characterizations

Millimeter-sized α-RuCl_3_ crystals were grown by the sublimation of RuCl_3_ powder sealed in a quartz tube under vacuum^52^. The growth was performed in a box furnace. After dwelling at 1060 °C for 6 h, the furnace was cooled to 800 °C at 4 °C/h. Magnetic order was confirmed to occur at 7 K by measuring magnetic properties and specific heat^21^.

### Inelastic X-ray scattering

The experiments were conducted at beam line 30-ID-C (HERIX) at the Advanced Photon Source (APS). The highly monochromatic X-ray beam of incident energy *E*_i_ = 23.7 keV (l = 0.5226 Å) was focused on the sample with a beam cross section of ∼35 × 15 mm^2^ (horizontal × vertical). The total energy resolution of the monochromatic X-ray beam and analyzer crystals was ΔE ∼1.3 meV (full width at half maximum). The measurements were performed in transmission geometry. Typical counting times were in the range of 30–120 s per point in the energy scans at constant momentum transfer **Q**. H, K, L are defined in the trigonal structure with a = b = 5.9639 Å, c = 17.17 Å at the room temperature.

### Density functional theory calculations of phonon spectrum

Phonon dispersions for α-RuCl_3_ were calculated using with density functional perturbation theory (DFPT) and the Vienna Ab initio Simulation Package (VASP). The exchange-correlation potential was treated within the generalized gradient approximation (GGA) of the Perdew-Burke-Ernzerhof variety, where the kinetic energy cutoff was set to 400 eV. Integration for the Brillouin zone was done by using a Monkhorst-Pack *k*-point grids which is equivalent to 8 × 8 × 9.

## Supplementary information

Supplementary Information

Peer Review File

## Data Availability

The data that support the findings of this study are available from the corresponding author on reasonable request.
