# Supplementary material for: Giant phonon anomalies in the proximate Kitaev quantum spin liquid α-RuCl3
Source: Nat Commun. 2021 Jun 10;12:3513. doi: 10.1038/s41467-021-23826-1 (PMC8192767; doi:10.1038/s41467-021-23826-1)
Supplement: Supplementary file 1 — Supplementary Information [file 41467_2021_23826_MOESM1_ESM.pdf]

Supplementary Information for

Giant Phonon Anomalies in the Proximate Kitaev Quantum Spin Liquid α-$RuCl_3$

Haoxiang Li[1], T. T. Zhang[2,3], A. Said[4], G. Fabbris[4], D. G. Mazzone[5,6], J. Q. Yan[1,7], D. Mandrus[1,7], G. Halasz[1], S. Okamoto[1], S. Murakami[2,3], M. P. M. Dean[5], H. N. Lee[1] and H. Miao[1,†]

[1]Material Science and Technology Division, Oak Ridge National Laboratory, Oak Ridge, Tennessee 37831, USA

[2]Department of Physics, Tokyo Institute of Technology, Okayama, Meguro-ku, Tokyo 152-8551, Japan

[3]Tokodai Institute for Element Strategy, Tokyo Institute of Technology, Nagatsuta, Midori-ku, Yokohama, Kanagawa 226-8503, Japan

[4]Advanced Photon Source, Argonne National Laboratory, Argonne, Illinois 60439, USA

[5]Condensed Matter Physics and Materials Science Department, Brookhaven National Laboratory, Upton, New York 11973, USA

[6]Laboratory for Neutron Scattering and Imaging, Paul Scherrer Institut, CH-5232 Villigen, Switzerland

[7]Department of Materials Science and Engineering, the University of Tennessee at Knoxville, Knoxville, Tennessee 37996, USA

†Correspondence should be addressed to miaoh@ornl.gov.

Supplementary Note 1: Inelastic X-ray scattering

Inelastic X-ray (IXS) scattering is powerful technique to study phonon excitations with meV energy resolution[1]. In our study, the experimental setup provides an energy resolution of 1.3 meV and 35×15 μm^2 spot size. The energy resolution is calibrated by fitting the elastic peak (Supplementary Fig. 1) to a pseudo-voigt function:

$$R(\omega) = (1-\alpha)\frac{I}{\sqrt{2\pi}\sigma}e^{-\frac{\omega}{2\sigma^2}} + \alpha\frac{I}{\pi}\frac{\Gamma}{\omega^2+\Gamma^2} \quad (1)$$

where the energy resolution is the full-width-at-half-maximum (FWHM).

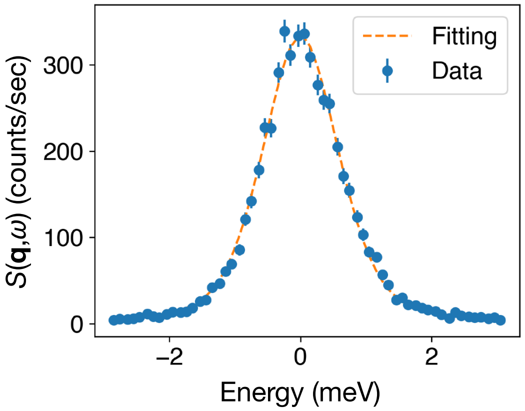

Supplementary Figure 1: IXS spectrum at Q = (6, -3, 0). The orange dashed line is a pseudo-voigt function fitting of the experimental data at the room temperature. The extracted energy resolution using the full width half maximum (FWHM) is ~1.3meV. The error bars represent one standard deviation assuming Poisson counting statistics.

IXS directly probes the phonon dynamical structure factor, $S(\mathbf{Q},\omega)$. The IXS cross-section for solid angle $d\Omega$ and bandwidth $d\omega$ can be expressed as[1]:

$$\frac{d^2\sigma}{d\Omega d\omega} = \frac{k_f}{k_i} r_0^2 |\vec{\epsilon_i} \cdot \vec{\epsilon_f}|^2 S(\mathbf{Q},\omega) \tag{2}$$

where $\mathbf{k}$ and $\boldsymbol{\epsilon}$ represent the scattering vector and x-ray polarization and i and f denote initial and final states. r_0 is the classical radius of the electron. In a typical measurement, the energy transfer ω is much smaller than the incident photon energy (23.71 keV in our study). Therefore, the term $\frac{\boldsymbol{k_f}}{\boldsymbol{k_i}} \sim 1$, and $\frac{d^2\sigma}{d\Omega d\omega} \propto S(\mathbf{Q},\omega)$.

$S(\mathbf{Q},\omega)$ is related to the imaginary part of the dynamical susceptibility, $\chi''(\mathbf{Q},\omega)$, through the fluctuation-dissipation theorem[1]:

$$S(\mathbf{Q},\omega) = \frac{1}{\pi} \frac{1}{(1-e^{\omega/k_B T})} \chi''(\mathbf{Q},\omega) \tag{3}$$

Where $\chi''(\mathbf{Q},\omega)$ can be described by the damped harmonic oscillator form[1], which has

antisymmetric Lorentzian lineshape:

$$\chi''(\mathbf{Q},\omega)=\sum_i I_i\left[\frac{\Gamma_i}{(\omega-\omega_{Q,i})^2+\Gamma_i^2}-\frac{\Gamma_i}{(\omega+\omega_{Q,i})^2+\Gamma_i^2}\right] \quad (4)$$

here i indexes the different phonon peaks.

Supplementary Note 2: Spectral peak fittings

The phonon peak can be extracted by fitting the IXS spectrum at constant-momentum transfer $\mathbf{Q}$, using Supplementary Eq. (3) and (4). Due to the finite experimental resolution, the IXS intensity is a convolution of $S(\mathbf{Q},\omega)$ and the instrumental resolution function, $R(\omega)$:

$$I(\mathbf{Q},\omega)=S(\mathbf{Q},\omega)\otimes R(\omega) \quad (5)$$

Here $R(\omega)$ was determined by fitting elastic peak near a Bragg condition (Supplementary Fig. 1).

The raw data of Fig. 2a in the main text is shown in Supplementary Fig. 2. The dashed curves represent fits of the experimental data following Supplementary Eq. (3)-(5). In all these fittings, the intrinsic phonon linewidth is much smaller than the energy resolution ΔE~1.3meV. Therefore, all peak-widths are essentially resolution-limited. Near the Bragg condition, an additional resolution-limited elastic peak is included to account for the elastic diffuse scattering. The extracted peak positions are shown in Fig. 2b of the main text. Supplementary Fig. 3 and Fig. 4 show the IXS raw data along the M-Γ_2 trajectory at 10 and 80 K, respectively. The extracted peak positions are shown in Fig. 2e of the main text.

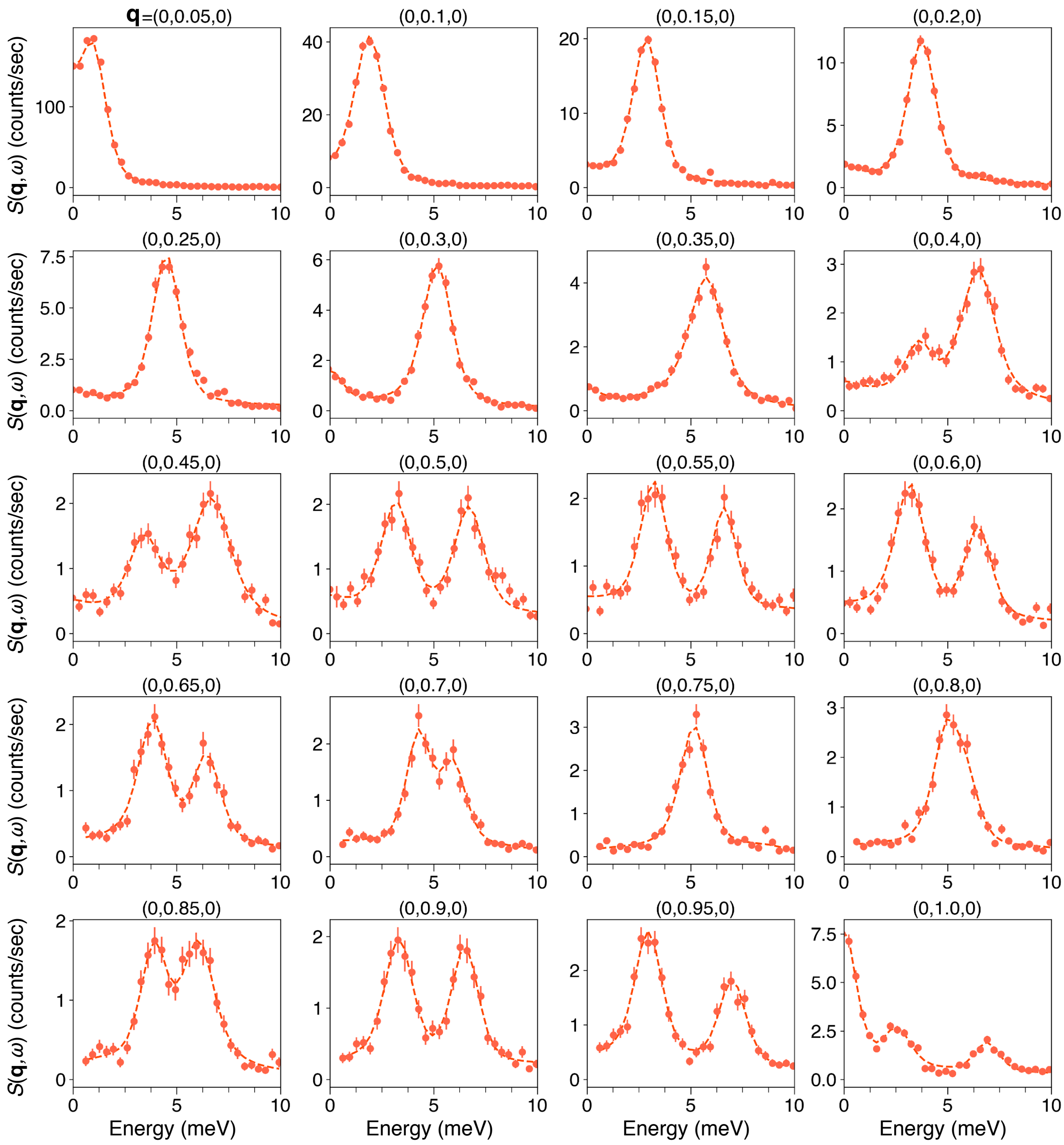

Supplementary Figure 2: IXS raw data along the G₁-M-G₂ direction at room temperature. Dashed curves are fittings based on Supplementary Eq. (3)-(5). Same as Fig. 3 in the main text, we define **q**=(0,0,0) and (0,1,0) as Γ_1 and Γ_2 respectively, and the M point is at **q**=(0,0.5,0). The extracted peak dispersions are shown in Fig. 2b in the main text. The error bars represent one standard deviation using Poisson counting statistics.

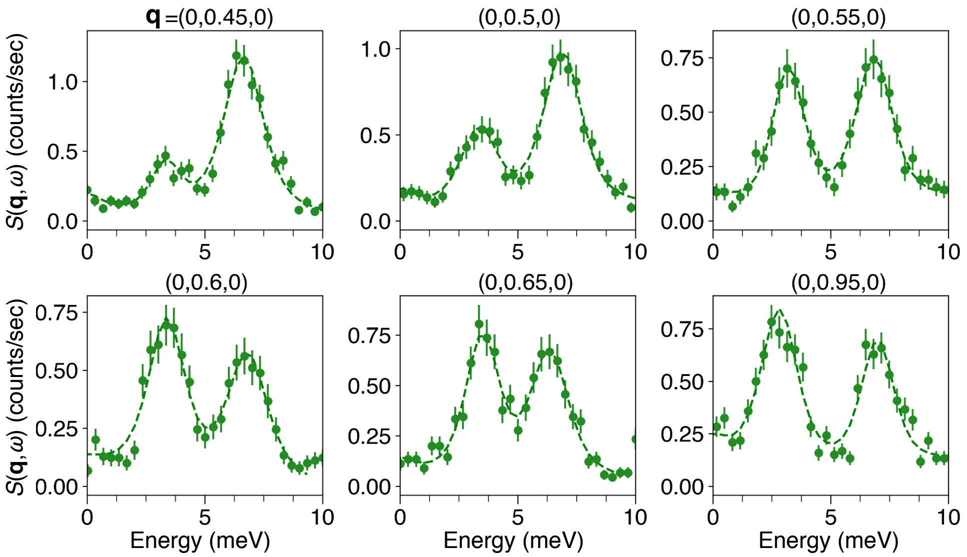

Supplementary Figure 3: IXS raw data along the M-Γ_2 trajectory at *T*=80 K. Dashed curves are fittings based on Supplementary Eq. (3)-(5). The error bars represent one standard deviation using Poisson statistics.

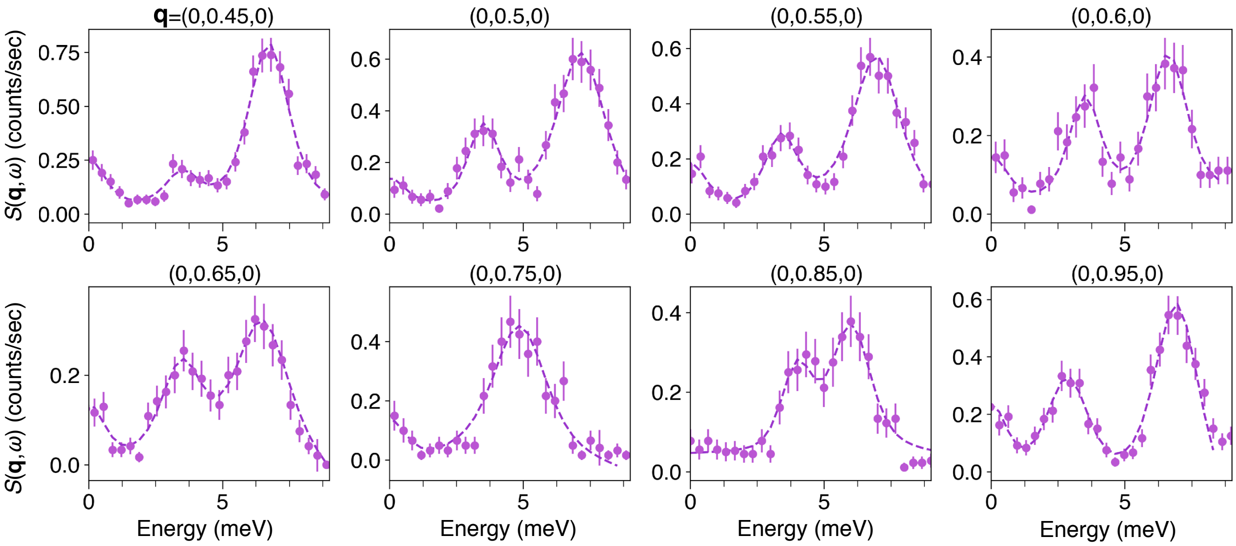

Supplementary Figure 4: IXS raw data along the M-Γ_2 trajectory at *T*=10 K. Dashed curves are fittings based on Supplementary Eq. (3)-(5). The error bars represent one standard deviation using Poisson statistics.

Supplementary Note 3: Additional information on the acoustic phonon softening

In this section, we show the temperature dependent IXS raw data at **q**=(0,0.05,0), (0,0.1,0) and (0,0.15,0) r.l.u. in Supplementary Fig. 5. In the first column of Supplementary Fig. 5a, the inelastic peaks at **q**=(0,0.05,0) r.l.u. are contaminated by the residual intensity from elastic scattering, especially for the spectrum at lowest temperature (T=10 K) where the thermal Bose factor greatly suppresses the count rate of the inelastic scattering (top left plot in panel a). Thus, the extracted peak position at T=10 K shown in Supplementary Fig. 5b have much larger error bar. On the other hand, In Supplementary Fig. 5c, the acoustic phonon peak at **q**=(0,0.2,0) r.l.u. and ω~4 meV does not show any phonon softening within the experimental uncertainty. This is in agreement with the data at **q**=(0,0.15,0) r.l.u. shown in the main text.

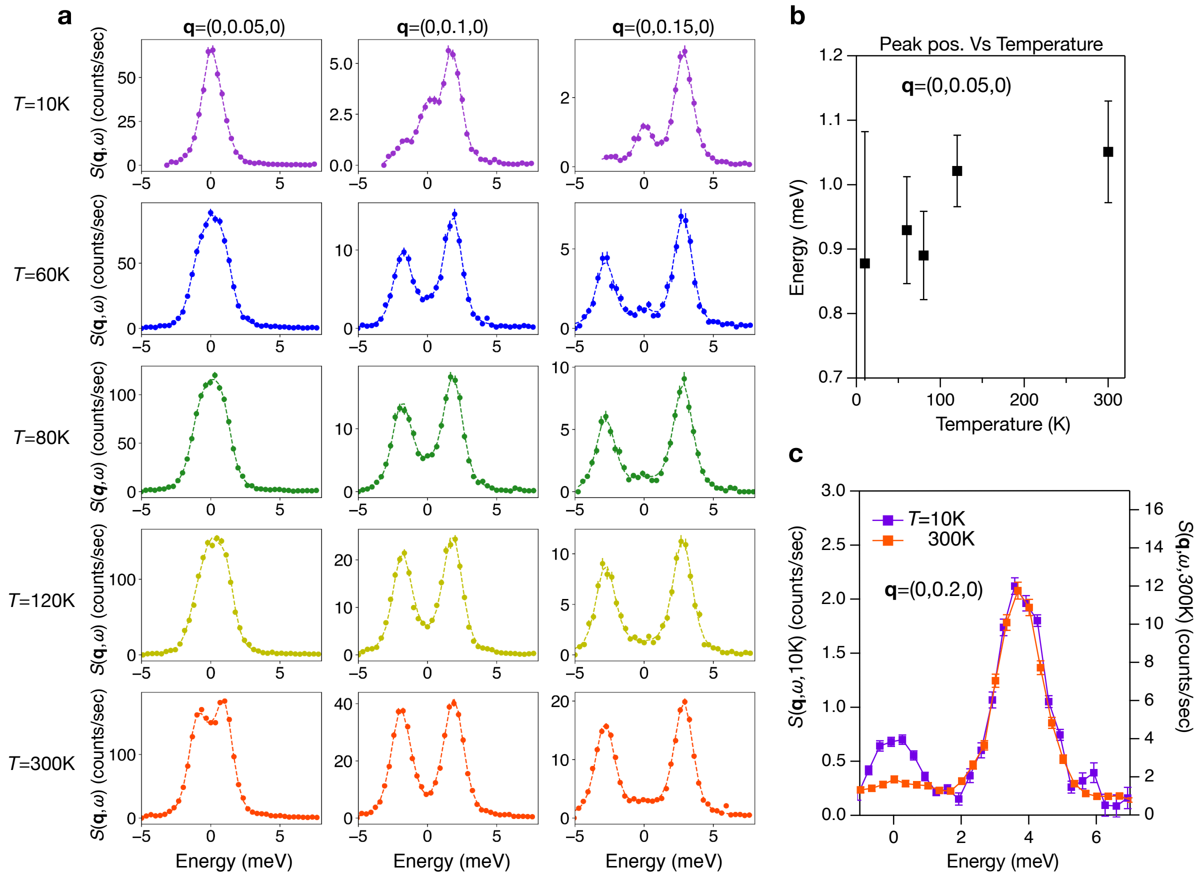

Supplementary Figure 5: (**a**) Temperature dependent IXS raw data of the acoustic phonon near Γ_1 (**q**=(0, 0.05, 0), (0, 0.1, 0) and (0, 0.15, 0)). Dashed curves are fittings based on Supplementary Eq. (3)-(5). (**b**) The inelastic peak positions vs temperature at **q**=(0,0.05,0) (**q**$_3$ in the main text), extracted from the fittings. At low temperature 10K, the IXS signal is dominated by the elastic intensity (see the raw data in panel **a**), thus the fit return a much larger error bar compared to that in higher temperature. (**c**) Raw IXS spectra at **q**=(0,0.2,0) with the acoustic phonon peak at ~4meV. The phonon peaks do not show softening effect. The error bars in panel **a** and **c** represent one standard deviation assuming Poisson counting statistics. The error bars in panel **b** denote the 2σ returned from the fittings that extract the spectral peak positions.

Supplementary Note 4: Additional information on the optical phonon spectral weight enhancement

As we show in the main text, the effect of spectral weight enhancement is larger as $\omega \to J_K$. However, as the interlaced optical phonons disperse in the (3, 7) meV energy window, we cannot examine the effect of spectral weight enhancement for $\omega > 7$ meV along the Γ-M direction. To estimate the upper energy scale of the effect we show the Bose-factor corrected IXS data at **q**=(-0.35, 0.65, 0.09) in Supplementary Fig. 6, which is slightly away from the high symmetry point K at **q**=(-0.33, 0.67, 0). This data is collected by a different analyzer with a smaller 2θ than the main detector. The phonon peak at $\omega \to 11$ meV does not show spectral weight enhancement. These results suggest that the effect of spectral weight enhancement is the largest near $\omega \sim J_{\mathrm{K}}$.

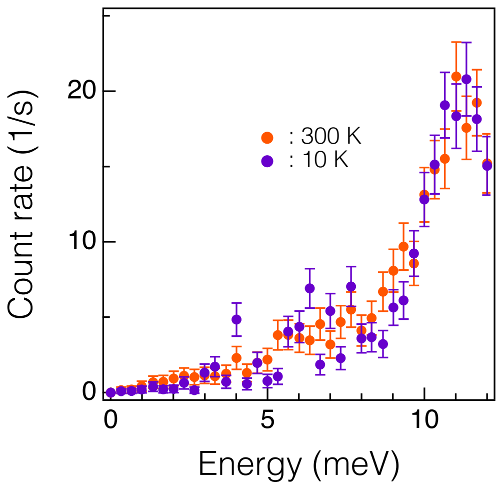

Supplementary Figure 6: Bose-factor corrected IXS data at **q**=(-0.35, 0.65, 0.09). This **q** position is close to the high symmetry point K at **q**=(-0.33, 0.67, 0). Purple and orange marks represent data at 10 and 300 K, respectively. Error bars represent one standard deviation from Poisson statistics.

Supplementary Note 5: Effects of structural transition

α-$RuCl_3$ has a first order structural phase transition around 150 K with large thermal hysteresis[2]. Below the structural phase transition, both the stacking of the honeycomb layer and the Ru-Cl-Ru bond angle are modified. Previous experimental studies indicate that the low-temperature structure enhances the Kitaev term[3-15] and hence strengthens the coupling of phonon to fraction excitations in α-$RuCl_3$. This is consistent with our observation of two type phonon anomalies at characteristic Kitaev energy and temperature scales.

Supplementary Note 6: DFT calculation of the phonon dispersion

In this section, we show the density functional theory (DFT) calculated phonon dispersion of α-$RuCl_3$. Due to the week inter-layer van der Waals interaction, α-$RuCl_3$ displays stacking-faults that hinder a conclusive determination of the crystal structure. Early powder diffraction study suggested that both a trigonal space group $P3_112$ with 3-layer stacking periodicity (Supplementary

Fig. 7a and b) and a monoclinic space group $C2/\mathrm{m}$ stacking (Supplementary Fig. 7c and d) are consistent with the experimental data[5]. More recent x-ray and neutron studies of α-$RuCl_3$ single crystals support space group $C2/\mathrm{m}$[16,17]. This is consistent with our DFT calculations of the nonmagnetic phase, where we observe large negative energy phonon branches in space group $P3_112$ (Supplementary Fig. 7e), suggesting an unstable crystal structure. In contrast, the phonon energy is positive under space group $C2/\mathrm{m}$ except for one transverse acoustic mode along the Γ-Z direction (see Supplementary Figure 7f), consistent with the presence of stacking-faults. We thus choose space group $C2/\mathrm{m}$ for our calculations.

In Supplementary Fig. 7f, we compare the sinusoidal fitted phonon modes along the Γ-M direction (dashed blue curves) with the DFT calculations. While the in-plane transverse acoustic phonon agrees well with the calculation, DFT fails to reproduce the interlaced optical phonons. As we discussed in the main text, α-$RuCl_3$ has zigzag magnetic order below T_N~7 K, while above T_N it is a paramagnetic Mott insulator with large Mott gap that persists up to room temperature[18,19]. To capture these effects, we performed DFT+SOC+U calculations in the nonmagnetic and zigzag magnetic phase, using experimentally determined lattice and magnetic parameters[2]. Here U=4.0 eV and J_H=-0.5eV are consistent with a previous study[17]. As shown in Supplementary Fig. 8a and b, the nonmagnetic phase remains metallic while the magnetic phase opens a Mott gap in their electronic structure. However, the calculated phonon dispersion displays large negative frequencies. These results suggest that the ground state wave function is strongly modified by the large magnetic fluctuations that are not captured by our DFT+SOC+U scheme. More sophisticated calculations are required to properly describe the electronic and lattice dynamics of α-$RuCl_3$.

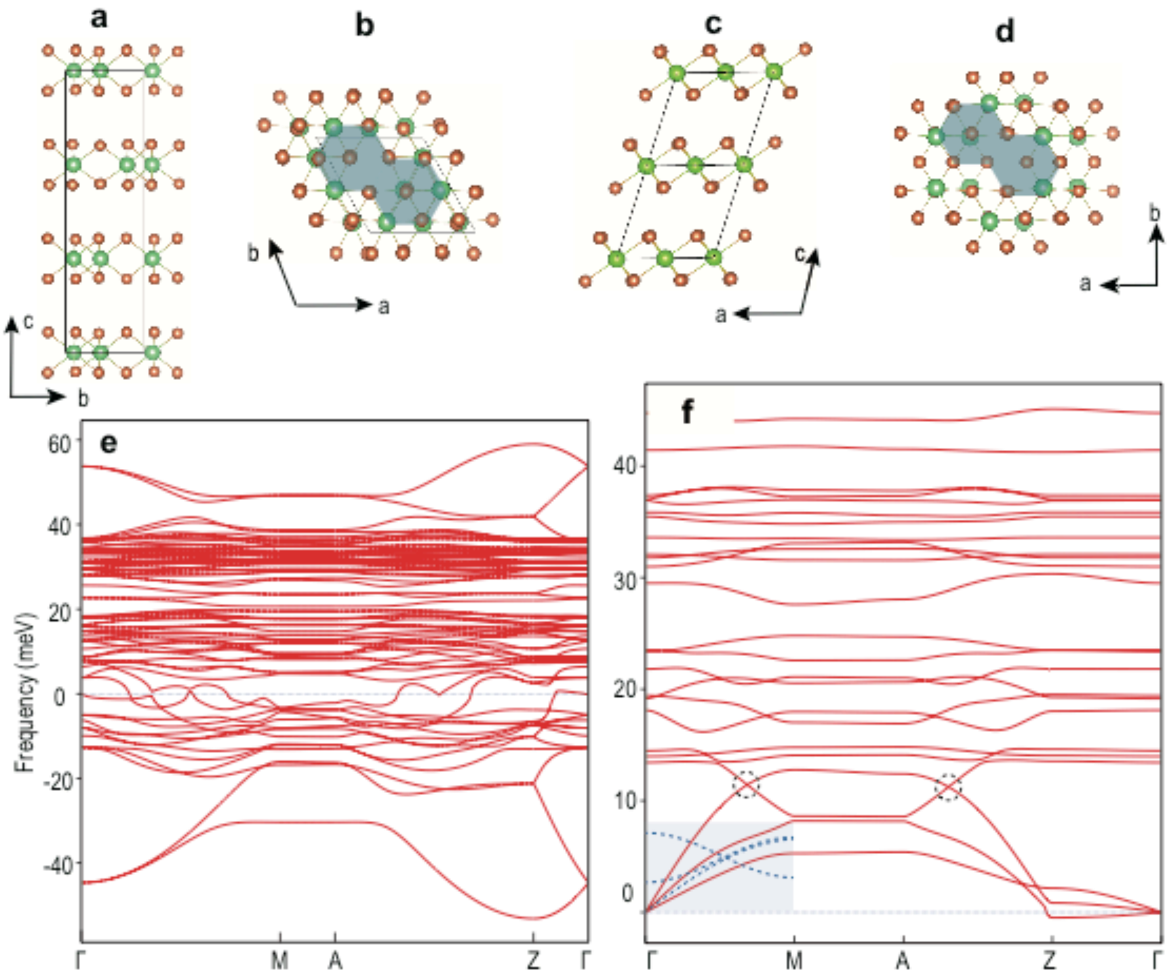

Supplementary Figure 7: DFT calculation of the phonon dispersion. (a) and (b) are the side and top view crystal structure of α-$RuCl_3$ with a $P3_112$ space group. (c) and (d) show the crystal structure with a C2/m space group. (e) and (f) are the DFT calculated phonon dispersion under $P3_112$ and C2/m, respectively. The blue dashed curves in (f) are sinusoidal fitting of experimental data.

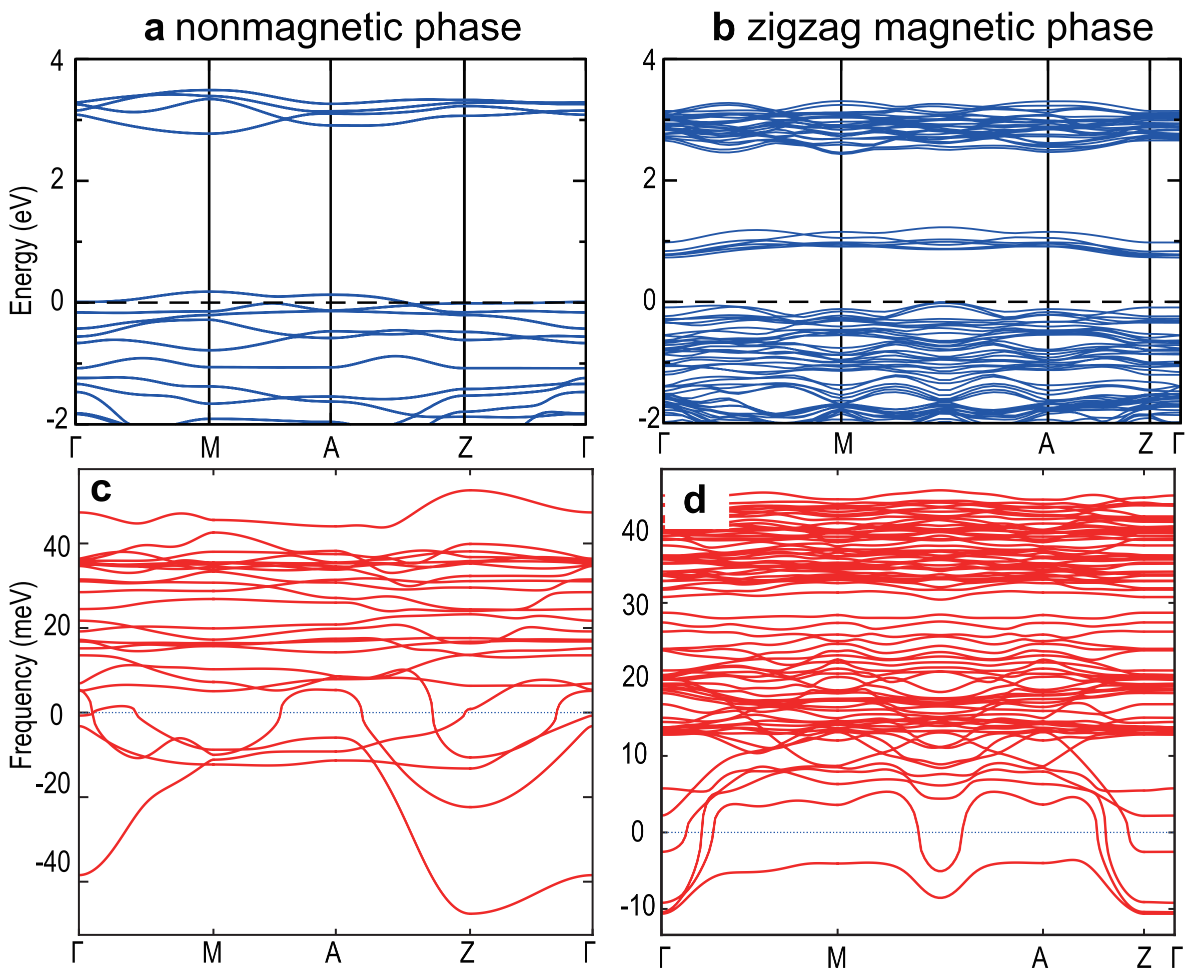

Supplementary Figure 8: DFT+SOC+U calculations. (a) and (c) show the electronic and phonon dispersion of α-$RuCl_3$ in the nonmagnetic phase. (b) and (d) are the same plots but for the zigzag magnetic phase. Lattice parameters and magnetic moments are taken from ref. 16. U=4.0 eV, J_H=-0.5 eV, and soc are considered in all the calculations.

Supplementary Note 7: Topological nodal lines

While our DFT calculations failed to reproduce the low energy interlaced optical phonons, we find similar gapless phonon band-crossings at higher energy (~12 meV). Here we discuss the topological origin of these phonon bands. In the nonmagnetic phase, the *PT*-symmetry (where *P* and *T* are the inversion is time-reversal symmetry respectively) enforces gapless Weyl-points into nodal-lines (dashed circles in in Supplementary Fig. 7f). Based on the topological phonon

diagnosis algorithm[20], we find that the compatibility condition is not satisfied for space group *C*2/m (No.12). We thus use the symmetry-based indicator (SBI) of space group No.2, $Z_2Z_2Z_2Z_4$, which is the only subgroup of the original space group No. 12 that satisfies both compatibility condition and nontrivial SBI. The indicator of the lowest bands in Supplementary Fig. 7f are $Z_2Z_2Z_2Z_4$=(0000), corresponding to 0 mod 4 nodal lines in the Brillouin zone. A detailed first-principle calculation shows that there are 16 endless nodal lines heading to the [001] direction, where 6 of them are pinned onto the mirror plane.

Supplementary Note 8: Momentum dependent spectral enhancement and extracted peak widths

Supplementary Figure 9 shows the difference in the integrated phonon spectral weight between 10 and 300 K as function of momentum, revealing the intensity enhancement around the high symmetry point, consistent with the prediction from ref. 21. On the other hand, **q**=(0,0.45,0) shows larger enhancement compared to the other **q**. As we discussed in the main text, the transverse acoustic phonon that merge with the optical phonon near the M point may be the cause of this stronger enhancement at q=0.45. At lower q, the spectral intensity shows minimal enhancement effect across the Kitaev temperature (Supplementary Fig. 10). Supplementary Fig. 11 shows the extracted peak widths of the optical branches, which are all well below the resolution limit, and the change of the widths between 300 K and 10 K are relatively small.

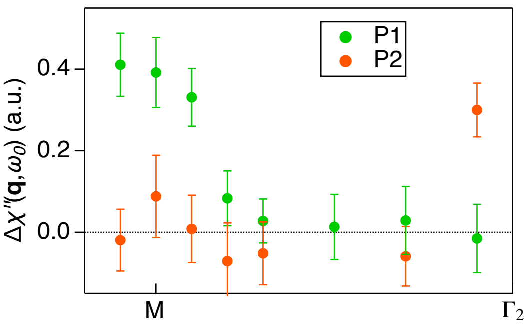

Supplementary Figure 9: q-dependent spectral enhancement. Error bars represent one standard deviation from Poisson statistics.

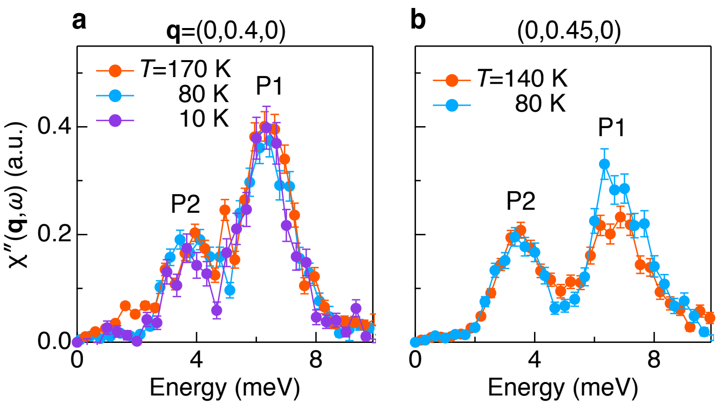

Supplementary Fig. 10: Spectral enhancement at q=0.4 and 0.45 from a different α-RuCl₃ sample. Error bars represent one standard deviation from Poisson statistics.

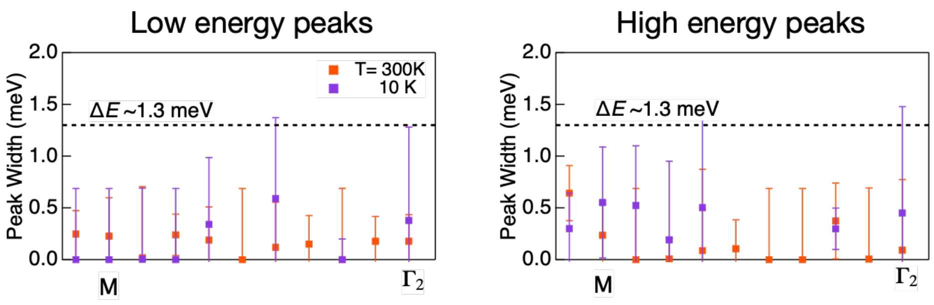

Supplementary Fig. 11: Extracted peak widths of the two interlaced optical phonon bands. The peak width value of the low energy (left panel) and the high energy (right panel) phonon peaks are all well below the energy resolution ΔE~1.3 meV (black dashed line). The error bars denote the 2σ returned from the fittings that extract the spectral peak widths.

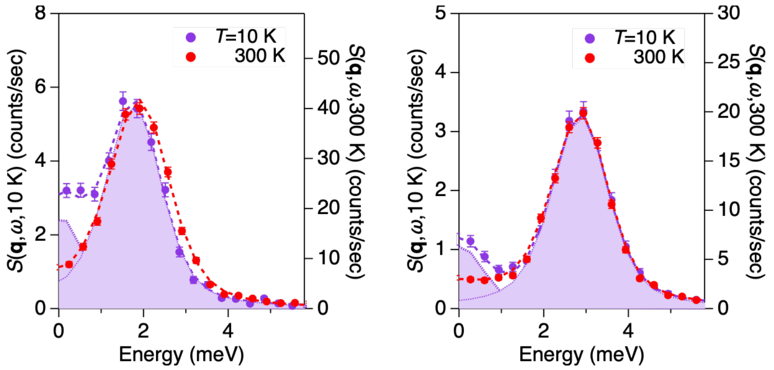

Supplementary Fig. 12: Same data shown in Fig. 4 c&d in the main text. Here we plot it with larger energy range and put the main peak in front to show the low energy tail. Error bars represent one standard deviation from Poisson statistics.

References:

1. Baron, A. Q. R. High-Resolution Inelastic X-Ray Scattering II: Scattering Theory, Harmonic Phonons, and Calculations. *Synchrotron Light Sources and Free-Electron Lasers* 1721–1757 (2016). doi:10.1007/978-3-319-14394-1_52.

2. R. D. Johnson et al., Monoclinic crystal structure of α-$RuCl_3$ and the zigzag antiferromagnetic ground state. Phys. Rev. B 92, 235119 (2015).
3. Sandilands, L. J., Tian, Y., Plumb, K. W., Kim, Y.-J. & Burch, K. S. Scattering Continuum and Possible Fractionalized Excitations in α−$RuCl_3$. *Phys. Rev. Lett.* **114**, 147201 (2015).
4. Nasu, J., Knolle, J., Kovrizhin, D. L., Motome, Y. & Moessner, R. Fermionic response from fractionalization in an insulating two-dimensional magnet. *Nature Physics* **12,** 912–915 (2016).
5. Banerjee, A. *et al.* Proximate Kitaev quantum spin liquid behaviour in a honeycomb magnet. *Nat. Mat.* **15,** 733–740 (2016).
6. Hermanns, M *et al*. Physics of the Kitaev model: fractionalization, dynamic correlations, and material connections. *Annu. Rev. Condens. Matter Phys*. **9**:17-33 (2017).
7. Banerjee, A. *et al.* Neutron scattering in the proximate quantum spin liquid α-$RuCl_3$. *Science* **356,**1055–1059 (2017).
8. Do, S.-H. *et al.* Majorana fermions in the Kitaev quantum spin system α -$RuCl_3$. *Nat. Phys.* **13,** 1079–1084 (2017).
9. Jansa, N. *et al*. Observation of two types of fractional excitation in the Kitaev honeycomb magnet. *Nat. Phys*. **14**, 786 (2018).
10. Widmann, S. *et al*. Thermal dynamic evidence of fractionalized excitations in α−$RuCl_3$. *Phys. Rev. B* **99**, 094415 (2019).
11. Broholm, C. et al., Quantum spin liquid. *Science* **367**, 263 (2020).
12. Takagi, H *et al*., Concept and realization of Kitaev quantum spin liquids. *Nat. Rev. Phys.* **1**, 246 (2019).

13. Kasahara, Y. *et al.* Majorana quantization and half-integer thermal quantum Hall effect in a Kitaev spin liquid. *Nature* **559,** 227–231 (2018).

14. Yamashita, M., Kurita, N. and Tanaka, H. Sample dependence of the half-integer quantized thermal Hall effect in the Kitaev candidate α−$RuCl_3$. arXiv:2005.00798 (2020).

15. Nasu, J., Yoshitake, J. & Motome, Y. Thermal Transport in the Kitaev Model. *Phys. Rev. Lett.* **119,** 127204 (2017).

16. H. B. Cao et al., Low-temperature crystal and magnetic structure of α-$RuCl_3$. *Phys. Rev. B* **93**, 134423 (2016).

17. Sinn, S., Kim, C., Kim, B. et al. Electronic Structure of the Kitaev Material α-$RuCl_3$ Probed by Photoemission and Inverse Photoemission Spectroscopies. *Sci Rep* **6**, 39544 (2016).

18. X. Q. Zhou *et al.*, Angle-resolved photoemission study of the Kitaev candidate α-$RuCl_3$, Phys. Rev. B 94, 161106 (2016).

19. H. B. Cao et al., Low-temperature crystal and magnetic structure of α-$RuCl_3$. *Phys. Rev. B* **93**, 134423 (2016).

20. Tiantian Zhang, et al. A diagnosis scheme for topological degeneracies at high-symmetry momenta. Phys. Rev. Research 2 (2), 2066 (2020).

21. Metavitsiadis, A and Brenig, W. Phonon renormalization in the Kitaev quantum spin liquid. *Phys. Rev. B* **101**, 035103 (2020).
