# Supplementary material for: Giant phonon anomalies in the proximate Kitaev quantum spin liquid α-RuCl3
Source: Nat Commun. 2021 Jun 10;12:3513. doi: 10.1038/s41467-021-23826-1 (PMC8192767; doi:10.1038/s41467-021-23826-1)
Supplement: Supplementary file 2 — Peer Review File [file 41467_2021_23826_MOESM2_ESM.pdf]

Editorial Note: This manuscript has been previously reviewed at another journal that is not operating a transparent peer review scheme. The manuscript was considered suitable for publication without further review at *Nature Communications.*
